# The Incidence and Electroencephalographic Features in Brain Tumor-Related Epilepsy

**DOI:** 10.3390/neurolint18090164

**Published:** 2026-08-26

**Authors:** Lejla Zonić, Renata Hodžić, Dževdet Smajlović, Larisa Kovačević, Samra Kadić-Vukas, Lejla Tandir-Lihić, Mirsad Hodžić

**Affiliations:** 1 Department for Neurology, University Clinical Centre Tuzla, 75000 Tuzla, Bosnia and Herzegovina; zonic.lejla@ukctuzla.ba (L.Z.); dzevdet.smailovic@ukctuzla.ba (D.S.); larisa.kovacevic@ukctuzla.ba (L.K.); 2 Department for Neurology, Cantonal Hospital Zenica, 72000 Zenica, Bosnia and Herzegovina; 3 Department for Neurosurgery, University Clinical Centre Tuzla, 75000 Tuzla, Bosnia and Herzegovina

**Keywords:** epileptic seizures, brain tumor-related epilepsy, electroencephalography

## Abstract

**Methods:** This retrospective–prospective study included 90 patients with BT. Based on the type of BT, the participants were divided into three groups of 30 patients each: patients with gliomas, with meningiomas and with brain metastasis. Demographic data such as age, sex, alcohol and cigarette consumption were collected. In all three study groups, the time of onset of the first ES, type, characteristics, and frequency were analyzed as well as abnormalities on electroencephalography (EEG). EEG was reviewed for any abnormal background or interictal epileptic discharges. **Results:** Brain tumor-related epilepsy (BTRE) was present in 46.7% of patients. The incidence of epilepsy secondary to brain tumors was 66.7% in patients with gliomas, 46.6% in those with meningiomas, and 26.7% in those with metastasis. The tumors associated with BTRE were most commonly located in the frontal (40%) and temporal (32.2%) lobes, followed by the parietal (25.6%) and occipital (2.2%) lobes. Focal to bilateral tonic–clonic ES represented the most common seizure type among the studied patients. Abnormalities on the initial EEG recordings were observed in 33 patients with BTRE. Focal EEG abnormalities were identified in 54.7% of patients, while diffuse changes were present in 23.8% of patients (*p* < 0.005). **Conclusions:** The highest incidence of BTRE was observed in patients with gliomas, followed by those with meningiomas, while the lowest incidence was recorded in the group with metastases. Smaller and slower growing tumors were associated with higher incidence of seizures than larger, more rapidly growing lesions. Focal to bilateral tonic–clonic seizures were the most common seizure type among patients with BTRE.

## 1. Introduction

Epileptic seizures (ES) are the most common symptom in patients with brain tumor (BT) and often represent the first clinical sign that leads to diagnosis. An epileptic seizure is conceptually defined as: ‘a transient occurrence of signs and/or symptoms due to abnormal excessive or synchronous neuronal activity in the brain’. Epilepsy is a brain disorder and is characterized by an abnormally increased predisposition to ES and is usually applied in clinical practice as having two unprovoked seizures, >24 h apart [1]. In 2014, the International League Against Epilepsy (ILAE) proposed the following practical clinical definition of epilepsy: ‘≥2 unprovoked (or reflex) seizures occurring >24 h apart; one unprovoked (or reflex) seizure and a probability of further seizures similar to the general recurrence risk (≥60%) after two unprovoked seizures, occurring over the next 10 years; or diagnosis of an epilepsy syndrome’ [2]. In cases of BT, the diagnosis of epilepsy is usually made after one seizure.

The risk of brain tumor-related epilepsy (BTRE) is highly dependent on tumor histology [3]. Patients with diffuse low-grade gliomas develop BTRE with seizures reported in approximately 80% of cases [4,5]. Epileptic seizures (ES) are also common among patients with glioblastoma, occurring in approximately 62–68% of cases [6,7], whereas their reported incidence in patients with meningiomas ranges from 40% to 47% [8,9]. Compared with primary brain tumors, seizures are less frequently observed in patients with brain metastases, with a reported incidence of approximately 10–15% [10,11]. Tumor location may also influence seizure susceptibility, as lesions involving the frontal, parietal, or temporal regions have been associated with a greater risk of seizures than those located in the occipital region [12,13]. Among patients with brain metastases, factors associated with an increased risk of BTRE include the presence of more than four metastatic lesions, involvement of the frontal, parietal, temporal, or occipital cortex, and melanoma as the primary malignancy [11]. Electroencephalography (EEG) plays an important role in the diagnostic evaluation and clinical management of patients with BTRE [14]. EEG findings may include focal or generalized slowing within the theta- or delta-frequency ranges, which can become more prominent during hyperventilation, as well as epileptiform discharges arising from the tumor or the surrounding peritumoral tissue [15]. Up to 10% of patients with a primary BT may show Periodic Discharges (PDs) [16,17], which refers to an EEG pattern characterized by a waveform that recurs at almost regular intervals with uniform morphology and duration and is spaced by a regular and discernible inter-discharge period. Based on the EEG localization, PDs are defined as generalized (GPDs) or lateraled (LPDs) [18]. Epileptic seizures, especially when uncontrolled, have a negative effect on quality of life and cognitive functioning in patients with BT [19]. This is the first study from Bosnia and Herzegovina addressing the incidence and EEG characteristics of BTRE in relation to different intracranial tumor types. We believe that the results may contribute to a better understanding of BTRE and support improved clinical decision-making in everyday practice.

The aim of this study was to assess the incidence and electroencephalographic features of epileptic seizures (ES) in patients with brain tumors (BT).

## 2. Material and Methods

### 2.1. Patients and Study Design

This retrospective–prospective study was conducted at the Clinic of Neurology and Clinic of Neurosurgery, University Clinical Centre Tuzla, Bosnia and Herzegovina during the period January 2024 to January 2026. During the first year of the study period (January 2024 to January 2025), data were collected retrospectively from the medical records of patients managed at the participating clinics. During the second year (January 2025 to January 2026), patients were enrolled and followed prospectively according to the study protocol. Patients from both study phases were identified according to the same predefined inclusion and exclusion criteria. Inclusion criteria were: adult patients who meet the following criteria diagnosis of a BT established with Computerized Tomography (CT) or Magnetic Resonance (MRI) of the brain, and subsequently confirmed after surgery by the histopathology findings of the Department of Pathology of the University Clinical Centre Tuzla; patients with Karnofsky Performance scale score over 70% and Eastern Cooperative Oncology Group (ECOG) score less than 2 and written informed consent for participation in the study provided by the patient or a close family member. Patients younger than 16 years, with Karnofsky score less than 70% and ECOG score over 2, as well as patients whose BT was not confirmed were excluded from the study. The same variables were collected for all study groups. Data from the retrospective and prospective cohorts were reviewed and harmonized to ensure consistency before statistical analysis. The study cohort consisted of three tumor groups: gliomas, meningiomas and brain metastasis/metastases. Thirty patients were included in each group. This sampling strategy was chosen to achieve balanced group sizes and facilitate comparative statistical analysis. Patients were enrolled consecutively during the defined study period, and no patients were selected based on seizure status or EEG findings. All patients received prophylactic levetiracetam administered twice daily, starting immediately after the diagnosis was confirmed and continuing until the end of the study follow-up period. Levetiracetam was used as a therapy in patients with preoperative seizures and as prophylactic treatment in patients without previous seizures. The same ASM was used across all study groups.

#### 2.1.1. Methods

Demographic data including age, gender, and alcohol and cigarette consumption were collected for all patients as well as clinical features, tumor characteristics (including histology, localization and size), and imaging findings. Gliomas were classified according to size into small (<3 cm), medium (3–5 cm), and large (>5 cm in the largest diameter). We followed the patients for six months after the surgical procedure in order to determine the occurrence of epileptic seizures and to assess their incidence as well as the seizure type. In all three groups of participants, we analyzed the time of occurrence of the first epileptic seizure (before and/or after surgical treatment), epileptic seizure type, duration, its features and incidence. Epileptic seizure type was determined according to the valid Classification of Epileptic Seizures adopted in 2017 by the ILAE [20], according to which seizures are classified into three main groups: 1. seizures with focal onset, which are divided into three sub types: (a) preserved or impaired awareness, (b) motor or non-motor onset, (c) focal to bilateral; 2. generalized seizures, which consist of motor or non-motor seizures; and 3. seizures of unknown onset may be motor or non-motor or unclassified [21]. The study received approval from the Ethics Committee from University Clinical Centre Tuzla and complied with the Declaration of Helsinki.

All patients underwent a standard electroencephalographic examination in the postoperative period and were repeated at the 6-month follow-up visit. Additional EEG recordings were performed when clinically indicated. Electroencephalographic activity was recorded using a high-resolution electroencephalographic system Medtronic Inc., (Mineapolis, MN, USA) type with 14 channels and two reference electrodes. Interpretation of EEG recordings were performed according to a uniform protocol that includes analysis of: baseline rhythm, baseline rhythm wave amplitude, “blocking response,” presence of asymmetries, tendency toward and/or presence of focal or paroxysmal discharges at rest and during provocation by hyperventilation and photo stimulation. The EEG recordings were classified as follows: 1. normal—a recording that does not deviate from physiological variations; 2. focal slowing—non-specific EEG abnormality indicating localized cortical dysfunction; 3. diffuse slowing—generalized cerebral dysfunction involving widespread brain regions; and 4. epileptic abnormalities. Epileptic abnormalities included spikes and sharp waves. Most of the EEGs were performed in the outpatient setting during scheduled follow-up visits, typically once every 3–6 months per year, in the context of ongoing seizure monitoring or neurological evaluation. Only a minority of recordings were conducted during hospitalizations. No continuous or ICU EEG monitoring was included. The average EEG recording duration was 20–30 minutes.

#### 2.1.2. Statistical Analysis

Numerical test results were statistically processed, analyzed, and compared in order to obtain answers to the questions formulated within the aims of the study. In the analysis of the final results, standard descriptive statistical methods were used, including measures of central tendency (mean and median) and measures of dispersion (range, percentiles, and standard deviation). All results were processed using Microsoft Office Excel 2007 and MedCalc Statistical Software 22.026 (MedCalC Software Ltd., Ostend, Belgium). *p* values less than or equal to 0.05 were considered statistically significant. Comparisons of categorical variables between groups were performed using the chi-square test. In cases where assumptions for the chi-square test were not met (i.e., low expected frequencies), the Fisher–Freeman–Halton exact test was used. Multi-variable binary logistic regression analysis was performed to identify independent predictors of BTRE. Odds ratios (OR) with 95% confidence intervals (CI) were calculated. A *p*-value < 0.05 was considered statistically significant.

## 3. Results

Group A included 30 patients with gliomas (63.3% female and 36.7% male; mean age 54.3 ± 14.8 years), Group B included 30 patients with meningeomas (46.7% female and 53.3% male; mean age 59.8 ± 12.1 years), and Group C included 30 patients with metastasis (36.7% female and 63.3% male; mean age 64.7 ± 7.2). Within the studied groups, 36 patients (40.0%) reported alcohol consumption, whereas 44 patients (48.9%) were cigarette smokers. In Group A, 10 of 30 patients (33.3%) consumed alcohol and 18 (60.0%) were cigarette smokers. In Group B, alcohol consumption was reported in 14 of 30 patients (46.7%), while 22 (73.3%) were smokers. In Group C, 12 of 30 patients (40.0%) consumed alcohol and 24 (80.0%) smoked cigarettes (Table 1). Age showed a non-significant trend toward increase across groups (ANOVA: F(2,87) = 3.05, *p* = 0.053). No significant differences were observed in sex distribution (χ^2^(2) = 3.74, *p* = 0.15), alcohol consumption (χ^2^(2) = 1.11, *p* = 0.57), or smoking status (χ^2^(2) = 3.05, *p* = 0.22). The frontal region was the most common tumor location (36/90, 40.0%), followed by temporal lobe (29/90, 32.2%), parietal (23/90, 25.6%), and occipital lobe (2/90, 2.2%), localization (Table 2). Tumor was right-sided in 31 patients (34.4%) and left-sided in 52 patients (57.8%), while bilateral involvement of both hemispheres was present in seven patients (7.8%). No statistically significant difference in tumor location (frontal, parietal, temporal, and occipital) was observed among the three study groups (*p* = 0.44), with frontal and temporal lobes being the most frequently affected regions across all groups. Histopathologically, in Group A, which comprised 30 patients with gliomas, 11 patients (36.7%) had low-grade tumors, while 19 patients (63.3%) had high-grade tumors. Tumor size distribution showed that 15 patients (50.0%) had medium-sized gliomas (3–5 cm), 10 (33.3%) had large tumors (>5 cm), and five (16.7%) had small tumors (<3 cm). Perifocal edema was verified in 80 of 90 patients (88.9%), including all patients in Groups A and C, and 20 patients (66.7%) in Group B. A statistically significant difference in perifocal edema was observed among the study groups (Fisher–Freeman–Halton test, *p* = 0.001), with universal involvement in Groups A and C and a lower prevalence in Group B. Patients with metastatic brain tumors included patients with primary lung cancer in 18 cases (60.0%), breast cancer in six cases (20.0%), gastrointestinal cancer in four cases (13.3%), melanoma in one case (3.3%), and lymphoma in one case (3.3%). Out of the 90 patients included in the study, 42 patients (46.7%) had epileptic seizures: 20 patients in Group A, 14 patients in Group B, and eight patients in Group C. According to the histopathological characteristics of BT, the incidence of epileptic seizures was 66.7% in Group A, 46.6% in Group B, and 26.7% in the Group C. All patients with low-grade gliomas experienced epileptic seizures (11/11, 100%), whereas seizures were present in 9 of 19 patients (47.4%) with high-grade gliomas. This difference was statistically significant (Fisher’s exact test, *p* = 0.004). Seizures occurred in all patients with small and medium-sized gliomas (15/15, 100%), while only 5 of 15 patients (33.3%) with large gliomas experienced seizures. A statistically significant association was observed between smaller tumor size and higher seizure incidence (Fisher’s exact test, *p* = 0.002). Regarding tumor localization, in Group A patients with epileptic seizure, (*n* = 20), nine (45%) patients had frontal lobe tumors, seven (35%) had temporal lobe tumors, three (15%) had parietal lobe tumors, and one (5%) had occipital lobe tumors (5.0%). In Group B patients with BTRE, seven (50%) patients presented with frontal lobe tumors, four (28.6%) with temporal lobe tumors, two (14.3%) with parietal involvement, and one (7.1%) with occipital localization. In Group C, among eight patients with epileptic seizures, three (37.5%) patients had frontal tumor localization (37.5%), two (25%) had parietal involvement, and two (25%) had temporal localization. The overall incidence of epileptic seizures differed significantly among the study groups, occurring in 66.7% of Group A, 46.7% of Group B, and 26.7% of Group C (χ^2^(2) = 8.47, *p* = 0.014). Among patients with epileptic seizures, tumor localization did not differ significantly between groups (χ^2^(6) = 1.21, *p* = 0.98), with frontal and temporal regions being the most frequently affected. The classification of the epileptic seizures in studied groups was presented in Table 3. Focal to bilateral tonic–clonic seizures represented the most common seizure type among the studied patients (40% in group A, 35.7% in group B, and 37.5% in group C). No statistically significant difference in seizure sub-type distribution was observed among the three study groups (Fisher–Freeman–Halton exact test, *p* > 0.05). Epileptic seizures presented as the first symptom in 17 (85%) out of 20 patients with BRTE in Group A, in 6 (42.9%) out of 14 patients with BRTE in Group B, and in 2 (25%) out of 8 patients with BRTE in Group C, while in the remaining patients, seizures were diagnosed during or after surgical intervention. The proportion of patients in whom seizures represented the initial clinical manifestation differed significantly among the study groups (Fisher–Freeman–Halton exact test, *p* < 0.01).

**Figure 1:**
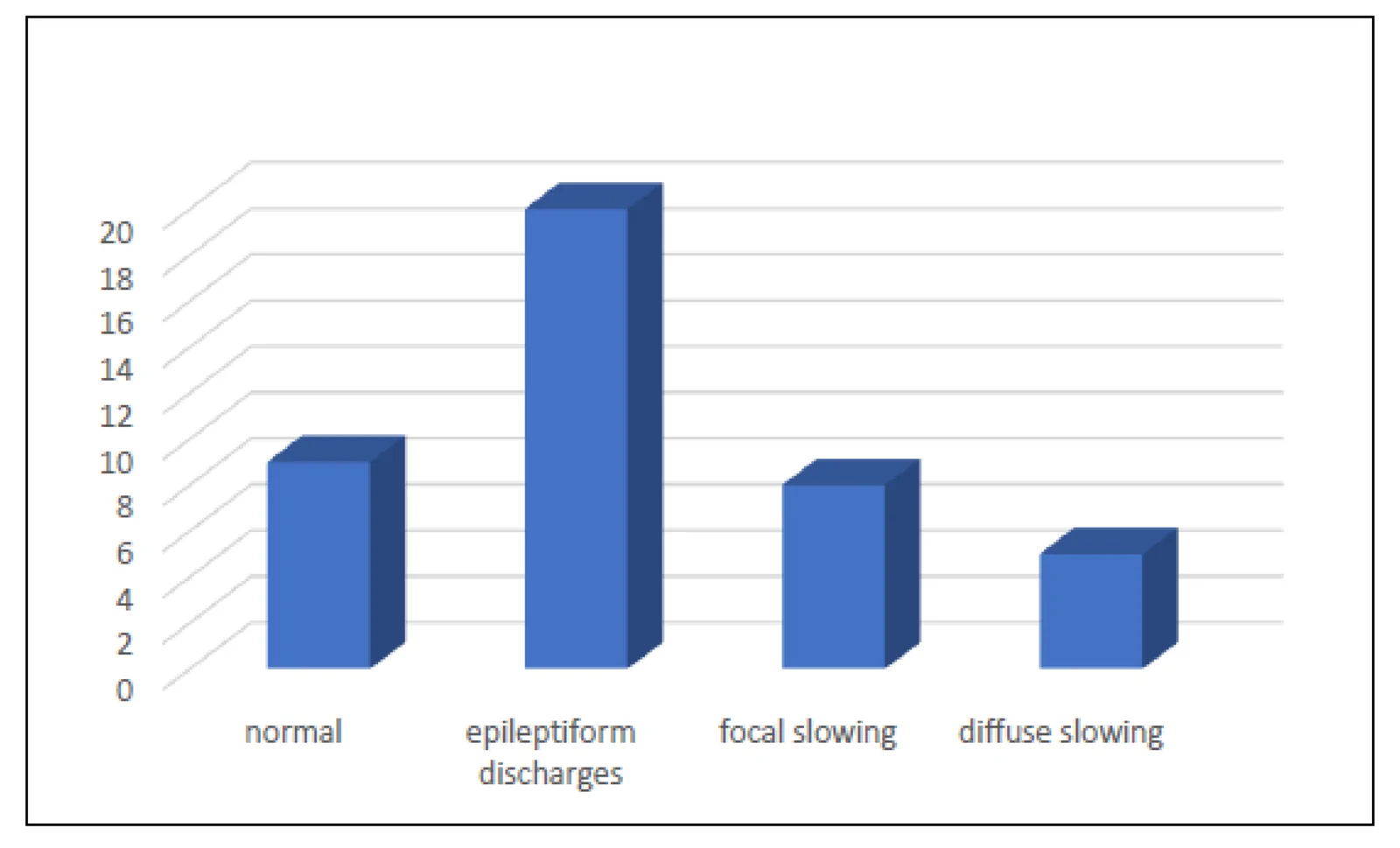
The electroencephalography findings in patients with brain tumor-related epilepsy.

Abnormal findings on the initial EEG recording were observed in 33 patients with BTRE. Non-epileptiform EEG abnormalities were observed in 13 patients (39.4%) and included focal slowing in eight patients (24.2%) and diffuse slowing in five patients (15.2%), reflecting regional or generalized cerebral dysfunction. Epileptic activity, consisting of spikes and sharp waves, was identified in 20 patients (60.6%), indicating increased cortical excitability. The distribution of EEG abnormality types differed significantly (χ^2^(2) = 10.36, *p* = 0.006). The distribution of interictal EEG abnormalities according to brain tumor type is presented in Figure 1. Multi-variable logistic regression analysis identified the study group and tumor location as independent predictors of BTRE. Patients in Group A had significantly higher odds of developing seizures compared with patients in Group C (OR = 3.42, 95% CI 1.52–7.71, *p* = 0.01). Temporal and frontal tumor localization were also associated with increased risk of BTRE (OR = 2.11, 95% CI 1.09–4.08, *p* = 0.02). Age showed a non-significant trend toward an increased risk of seizures (OR = 0.98, 95% CI 0.95–1.0, *p* = 0.18), whereas sex, smoking, and alcohol consumption were not significant predictors of BTRE.

## 4. Discussion

In our study, epileptic seizures (ES) were observed in 42 patients (46.7%): 20 patients (66.7%) in Group A, 14 patients (46.6%) in Group B, and 8 patients (26.7%) in Group C. The highest incidence of brain tumor-related epilepsy (BTRE) was recorded in patients with gliomas, followed by meningiomas, whereas the lowest incidence was observed in patients with brain metastases. Our findings demonstrate a significant association between smaller tumor size, lower histological grade, and higher incidence of epileptic seizures in patients with gliomas. The association between smaller tumor size and seizure occurrence observed in the glioma subgroup requires cautious interpretation. Several mechanisms may explain why smaller and lower-grade glioma are frequently associated with epilepsy. ES may represent an early clinical manifestation of tumor-related cortical dysfunction or slowly growing gliomas may induce prolonged interactions with the surrounding brain tissue, leading to cortical hyperexcitability through local inflammatory responses and changes in the extracellular environment. Regarding tumor localization in patients with BTRE, frontal lobe involvement was the most common finding across all study groups. In Group A, 45% of patients had frontal lobe tumors, followed by temporal (35%), parietal (15%), and occipital lobe localization (5%). In Group B, frontal localization was present in 50% of patients, temporal in 28.6%, parietal in 14.3%, and occipital in 7.1%. In Group C, frontal localization was observed in 37.5% of patients, while temporal and parietal localization were equally represented (25% each). The distribution of tumor localization was comparable across groups, with frontal and temporal regions being the most frequently affected. According to seizure semiology, focal to bilateral tonic–clonic seizures represented the most common seizure type among the studied patients, occurring in 40% of patients in Group A, 35.7% in Group B, and 37.5% in Group C. Initial EEG recordings demonstrated abnormalities in 33 patients with BTRE. EEG abnormalities included focal and diffuse changes. Focal abnormalities were the predominant pattern, whereas diffuse background abnormalities were less common. Epileptiform activity was observed in 60.6% of patients with abnormal EEG findings, followed by focal slowing (24.2%) and diffuse slowing (15.2%). Epileptiform discharges were significantly more frequent than non-epileptiform EEG abnormalities (*p* = 0.006). Epileptic seizures are among the most common symptoms in patients with brain tumors (BTs) and may represent the first clinical manifestation leading to diagnosis [22]. They occur as a result of disruption of normal neuronal electrical activity caused by tumor infiltration or compression of surrounding brain tissue. Epileptogenesis and tumorigenesis share several molecular, genetic, and cellular mechanisms and have therefore been described as “two sides of the same coin” [23]. Regardless of tumor location or histological type, the incidence of epileptic seizures in BTs ranges from 35% to 70%, while BTRE accounts for approximately 12% of acquired epilepsy and 4–10% of all epilepsy cases [24,25] Previous studies have shown that epileptic seizures are most frequently associated with gliomas, particularly low-grade gliomas located in superficial cortical or insular regions, where seizure incidence may reach 60–75%. Approximately 20–50% of patients with meningiomas and 20–35% of those with brain metastases also develop BTRE. Following tumor resection, approximately 60–90% of patients become seizure-free, with the most favorable outcomes reported in patients with glioneuronal tumors [26]. Our findings are consistent with these observations, particularly regarding the high incidence of BTRE in patients with gliomas and frontal or temporal tumor localization. Focal to bilateral tonic–clonic seizures have been reported as the predominant seizure type in patients with gliomas and meningiomas, whereas generalized tonic–clonic seizures are more commonly described in patients with brain metastases [26]. Similar findings were observed in our cohort. In our study, the tumor-related factors, particularly tumor location and study group (reflecting tumor biology), were the main independent predictors of BTRE, whereas patient-related factors such as sex, smoking, and alcohol consumption were not independently associated with seizure risk. This supports the hypothesis that epileptogenesis in BT patients is primarily driven by tumor histopathology rather than lifestyle-related factors. Electroencephalographic abnormalities are commonly present in patients with BTRE, particularly focal slowing, spikes, and sharp waves, which indicate cortical irritability and increased epileptogenicity [27,28]. Continuous focal delta slowing on EEG has been strongly associated with underlying structural lesions involving white matter tracts, further supporting the role of EEG in the evaluation of patients with BTRE. In the present study, all patients received prophylactic antiseizure medication (ASM) due to the high risk of seizures associated with BTs, particularly gliomas and cortical lesions. The use of levetiracetam in all may have influenced the observed seizure frequency. In patients with pre-existing seizures, levetiracetam represented therapeutic management, whereas in seizure-free patients it was administered as prophylaxis. Although the uniform use of the same ASM reduces variability related to treatment choice, current international guidelines, however, do not uniformly recommend routine prophylactic ASM in BT patients, given the lack of clear evidence for prevention of first seizures and the potential for adverse effects and drug interactions. Nevertheless, in clinical practice, prophylactic use remains common in many centers, particularly in high-risk cases involving cortical involvement or prior neurosurgical intervention. This institutional approach may have influenced seizure-related outcomes in our cohort and should be considered when interpreting the results. A limitation of this study is the relatively small sample size and short follow-up period of six months, which may have underestimated the incidence of late postoperative seizures and the long-term evolution of BTRE. EEG recordings were interpreted as part of routine clinical practice, and the reviewers were not blinded to the patients’ clinical characteristics, seizure status, tumor type, or imaging findings. Furthermore, routine EEG recordings were of short duration, and continuous EEG monitoring was not available, which may have limited the detection of intermittent epileptiform abnormalities.

In addition, the heterogeneity of tumor types may have influenced the assessment of seizure characteristics and outcomes. Future studies with larger patient cohorts and longer follow-up duration are needed to further validate these findings.

## 5. Conclusions

Among the three tumor groups, BTRE was most frequently observed in patients with gliomas, whereas lower rates were found in patients with meningiomas and brain metastases. The occurrence of seizures was associated with both the histological type and anatomical location of the tumor. Focal seizures with progression to bilateral tonic–clonic seizures represented the most frequently observed seizure pattern, while focal abnormalities were the predominant EEG finding. An association between tumor size and seizure occurrence was identified in the glioma subgroup but was not observed in patients with meningiomas or brain metastases.

## Figures and Tables

**Table 1:** Demographic data within the studied groups.

Variable	Group A	Group B	Group C
Number of patients	30	30	30
Age (mean ± SD) (years)	54.3 ± 14.8	59.8 ± 12.1	64.7 ± 7.2
Gender (M/F) (N)	11/19	16/14	19/11
Alcohol/cigarette consumption (N)	10/18	14/22	12/24

Group A, patients with gliomas; Group B, 30 patients with brain meningeomas; Group C, patients with brain metastasis/metastases.

**Table 2:** Localization of brain tumor within the studied groups.

Type of BT	Frontal n %	Parietal n %	Temporal n %	Occipital n %	Total n %
Group A	14 46.7	4 13.3	11 36.7	1 3.3	30 100
Group B	10 33.3	8 26.7	11 36.7	1 3.3	30 100
Group C	12 40.0	11 36.7	7 23.3	0 0.0	30 100
Total	36 40.0	23 25.6	29 32.2	2 2.2	90 100

Group A, patients with gliomas; Group B, 30 patients with brain meningiomas; Group C, patients with brain metastasis/metastases.

**Table 3:** Classification of the epileptic seizures in studied groups.

Epileptic Seizure Type	Group A n %	Group B n %	Group C n %
Focal aware seizures	5 25.0	4 28.6	1 12.5
Focal impaired awareness	6 30.0	4 28.6	1 12.5
Focal to bilateral tonic–clonic seiures	8 40.0	5 36.7	3 37.5
Generalized tonic–clonic seizures with bilateral onset	1 5.0	1 7.1	3 37.5
Total	20 100	14 100	8 100

Group A, patients with gliomas; Group B, 30 patients with brain meningiomas; Group C, patients with brain metastasis/metastases.

## Data Availability

Data available upon request from the authors.
